# Cigarette smoking is linked to an increased risk of delirium following arthroplasty in patients suffering from osteoarthritic pain

**DOI:** 10.1111/cns.14306

**Published:** 2023-06-19

**Authors:** Jie‐ru Chen, Jia‐qi Chen, Ji‐cheng Hu, Run‐sheng Huang, Liang Shen, Hai Gu, Xiao‐qing Chai, Di Wang

**Affiliations:** ^1^ Pain Clinic, Department of Anesthesiology, First Affiliated Hospital of USTC Division of Life Sciences and Medicine, University of Science and Technology of China Hefei China

**Keywords:** osteoarthritis, postoperative delirium, smoking, total knee arthroplasty

## Abstract

**Aims:**

Postoperative delirium (POD) is a common postoperative complication, and the potential relationship between cigarette smoking and POD is still unclear. The current study evaluated the relationship between preoperative smoking status in patients suffering from osteoarthritic pain and POD after total knee arthroplasty (TKA).

**Methods:**

A total of 254 patients who had undergone unilateral TKA were enrolled between November 2021 and December 2022, with no gender limitation. Preoperatively, patients' visual analog scale (VAS) scores at rest and during movement, hospital anxiety and depression (HAD) scores, pain catastrophizing scale (PCS) scores and smoking status were collected. The primary outcome was the incidence of POD, which was evaluated by the confusion assessment method (CAM).

**Results:**

A total of 188 patients had complete datasets for final analysis. POD was diagnosed in 41 of 188 patients (21.8%) who had complete data for analysis. The incidence of smoking was significantly higher in Group POD than in Group Non‐POD (22 of 41 patients [54%] vs. 47 of 147 patients [32%], *p* < 0.05). The postoperative hospital stays were also longer than those of Group Non‐POD (*p* < 0.001). Multiple logistic regression analysis showed that preoperative smoking (OR: 4.018, 95% CI: 1.158–13.947, *p* = 0.028) was a risk factor for the occurrence of POD in patients with TKA. The length of hospital stay was correlated with the occurrence of POD.

**Conclusions:**

Our findings suggest that patients who smoked preoperatively were at increased risk of developing POD following TKA.

## Graphical abstract

Delirium is a debilitating neuropsychological disorder in osteoarthritic patients after joint replacement surgery. Smoking and pain in patients with osteoarthritis are thought to interact in a positive feedback loop. Smoking has profound physiological effects on the neuropsychological system. This study found an association between smoking and postoperative delirium.

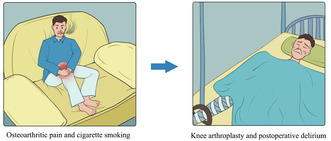

## 1 INTRODUCTION

Osteoarthritis (OA) is the most common joint disease that occurs frequently in the elderly and primarily affects knee joints, with an estimated 240 million adults worldwide suffering from symptomatic knee OA.^1^, ^2^ Pain is the prevailing symptom of OA and leads to limited physical function and disability. Total knee arthroplasty (TKA) is considered a cost‐effective therapy for end‐stage OA.^3^, ^4^ The demand for artificial total hip replacement and total knee replacement is expected to increase by 174 and 673% by 2030, respectively.^5^ Despite the increasing demand for joint replacement surgery, there are still many patients who suffer postoperative distress unrelated to the knee or hip itself, and the occurrence of postoperative complications such as postoperative delirium (POD) deserves our attention.^6^

Delirium is a state in which the patient experiences changes in consciousness, orientation, memory, perception, and behavior.^7^ POD is associated with poor prognosis in the perioperative period and increases the risk of long‐term cognitive impairment and reduces quality of life after surgery.^8^ Approximately 5–31% of patients experience POD after joint replacement surgery.^9^, ^10^ Unfortunately, the underlying pathogenesis mechanism of delirium is ambiguous. To optimize the postsurgical management of knee OA patients, it is of great significance to study the risk factors associated with POD. For example, demographic factors such as age and socioeconomic status as well as psychological factors such as anxiety/depression and catastrophizing have been identified as independent risk factors for predicting the incidence of POD following arthroplasty.^11^, ^12^, ^13^

Interestingly, the presence of smoking behavior is quite common in patients complicated with chronic pain, such as OA.^14^, ^15^ Among patients with OA, clinical findings estimated that one in five patients regularly smoke or use tobacco.^16^ Smoking has been implicated in the development of pain perception and pain experience in OA. Smokers have more severe pain than non‐smokers and are more than twice as likely as non‐smokers to have severe cartilage loss.^14^ In addition to pain experience, previous studies have illustrated that smoking causes neuroadaptations in the central nervous system; for example, cigarette smoking is associated with the psychopathology of anxiety/depression.^17^ There is also evidence that long‐term tobacco use increases the risk of developing anxiety symptoms and can exacerbate the severity of such symptoms.^18^ However, whether smoking in OA patients is related to POD incidence after TKA is unclear. In this study, we aimed to determine whether there is an association between smoking and POD in patients suffering from osteoarthritic pain.

## 2 MATERIALS AND METHODS

### 2.1 Study design

This is a prospective and observational study conducted at the First Affiliated Hospital of the University of Science and Technology of China (USTC). The study was conducted in accordance with the Declaration of Helsinki and approved by the Institutional Ethics Committee. All patients were informed of how the study protocol was carried out and signed an informed consent form.

### 2.2 Subjects

From November 2021 to December 2022, patients who were scheduled for TKA were assessed prior to the study. The inclusion criteria were as follows: patients aged from 18 to 65 years, body mass index (BMI) of 18–30 kg/m^2^, male or female, American Society of Anesthesiologists (ASA) class I–III, voluntary participation and the ability to complete all questionnaire assessments accurately.

Exclusion criteria were as follows: (1) patients unable to complete a baseline cognitive assessment or with a Mini‐Mental State Examination (MMSE) score < 24; (2) central nervous system (CNS) drugs were used preoperatively; (3) had sensory dysfunction in the past; (4) had a joint surgery on the affected joint; (5) had a revision surgery postoperatively; (6) preoperative hemoglobin reached the anemia standard and (7) researchers believed that other conditions did not qualify for this study, for example, patients with intraoperative bone cement syndrome and patients who had longer surgery duration than average.

### 2.3 General anesthesia

Prior to the induction of anesthesia, 40 mg methylprednisolone sodium succinate was administered. Midazolam injection 0.04 mg/kg, etomidate injection 0.2 mg/kg, sufentanil citrate injection 0.4 μg/kg and rocuronium bromide injection 0.8 mg/kg were administered in sequence. Intraoperatively, continuous intravenous infusion of propofol 4–6 mg/kg/h and remifentanil 6–10 μg/kg/h was used to maintain anesthesia.

All patients received general anesthesia, and they were given a femoral nerve block by the same staff after surgery. If the resting visual analog scale (VAS) pain score was >3 during postoperative hospitalization, additional flurbiprofen 50 mg was added for rescue analgesia. The anesthesiologist and the surgeon are the same.

### 2.4 Cigarette Smoking

The criteria for the assessment of smoking were:^19^, ^20^

*Current smokers*: current smoking or stopped smoking for <1 year.
*Former smokers*: smoking more than 10 packs a year but quitting smoking.
*Never smokers*: (1) people with no history of smoking and those who smoke <10 packs a year but stop smoking; (2) secondhand smoke (SHS): exposure to tobacco smoke from others at home or workplace for at least 15 min per day, 1 day per week and at least 2 years of the past 10 years.

### 2.5 Postoperative delirium

The confusion assessment method (CAM) delirium assessment tool is a widely used, delirium diagnostic scale developed by Inouye et al in the United States in 1990 for use by non‐psychiatrists. “The CAM diagnostic algorithm is based on four cardinal features of delirium: (1) acute fluctuating course, (2) attention disorder, (3) disturbance in thinking and (4) altered level of consciousness. According to CAM, the diagnosis of delirium requires the presence of features 1, 2 and 3 or 4”.^21^ The CAM scale has good sensitivity (94–100%) and specificity (90–95%), is short, easy to understand and use, and is therefore highly regarded by clinicians.^22^ POD is defined as any symptom of confusion that appears within 5 days of surgery.^7^, ^23^ “Delirium was assessed twice per day, between 06:00–08:00 and 18:00–20:00, with Chinese version of the CAM for non‐intubated patients and CAM‐ICU for intubated patients”.^24^ Patients who met the diagnostic criteria for CAM were classified as Group POD; otherwise, they were classified as Group Non‐POD.

### 2.6 Psychological variables

The hospital anxiety and depression (HAD) scale and pain catastrophizing (PCS) scale were also collected before TKA. HAD scored a total of 14 items, half of which were rated as depression and the other half as anxiety. Scores of 0 to 7 indicate no symptoms; scores of 8 to 10 indicate suspected anxiety/depression and scores of 11 to 21 indicate that anxiety/depression occurs definitely.^25^ If the PCS score is >30, the patient is considered to have pain catastrophizing.^1^

### 2.7 Outcome variables

#### 2.7.1 Primary outcome

The primary outcome was the incidence of POD.

#### 2.7.2 Other outcomes

Knee OA duration, duration of surgery, intraoperative blood loss, intraoperative midazolam, propofol, remifentanil and sufentanil dosage, postoperative rescue analgesia use, length of hospital stays, intensive care unit (ICU) admission, nausea/vomiting, blood transfusion, hypothermia and VAS scores were recorded for each patient.

### 2.8 Sample capacity

In our preliminary study, patients were grouped according to whether POD occurred after surgery. According to the study, the incidence of POD in the smoking population undergoing TKA was 44%. Furthermore, the overall incidence of POD was 19%, and the detection of significant differences between study groups was achieved by PASS 15.0 (NCSS, USA) software. Each group of 94 patients was required to provide 95% power with an alpha of 0.05. A 10% loss to follow‐up was taken into account, and we enrolled a total of 188 patients.

### 2.9 Statistical analysis

We performed all statistical analyses with SPSS version 26 (SPSS, Inc., an IBM Company, Chicago, IL, USA). The Kolmogorov–Smirnov test and Shapiro–Wilk test were used to evaluate the normality of the continuous data. Quantitative variables were shown as the mean values (with SD) or medians (with interquartile range) depending on (normally or skewed) the distribution of data, and categorical data were shown as percentages. The chi‐square test or Fisher's exact test was used to compare categorical data. Normal data were compared by independent sample *t‐*tests, and skewness comparisons were made using Mann–Whitney *U*‐tests. After univariable analysis, a binary logistic regression model was used for multivariable analysis. A two‐sided *p* value <0.05 was considered statistically significant.

## 3 RESULTS

### 3.1 Characteristics of patients with and without POD

**FIGURE 1:**
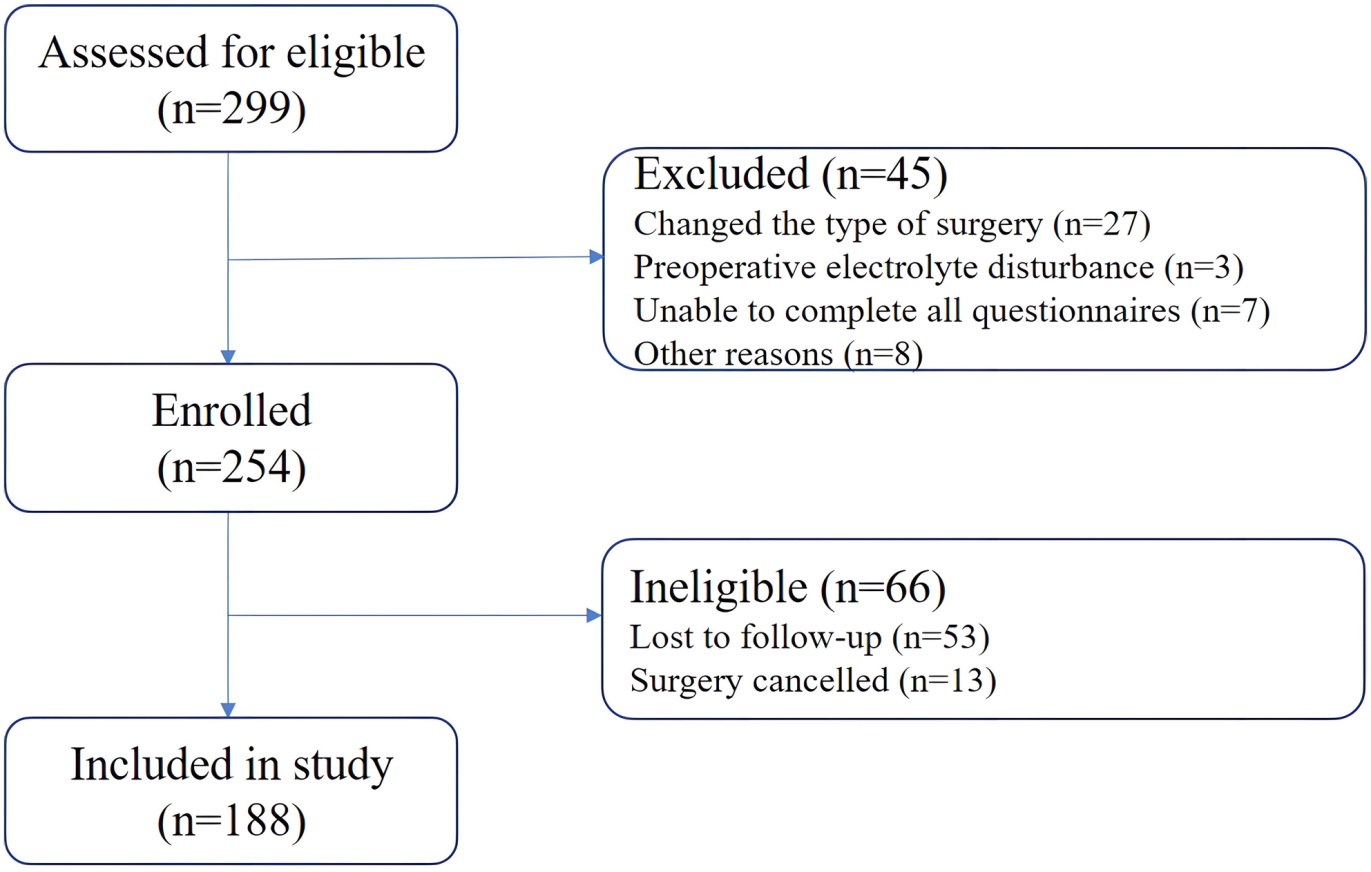
Flow diagram of the recruitment process.

Of the 299 patients who were assessed, 45 patients were ineligible due to changes in surgical types by surgeons and patients' own reasons, and 254 patients were qualified. Of these, 66 patients were excluded for the reasons described in the flow diagram, leaving 188 patients for the analysis (Figure 1). Patients were divided into POD and Non‐POD groups based on the results of the delirium assessment. Patient characteristics are presented in Table 1.

**TABLE 1:** General characteristics of patients in both groups (*n* = 188).

Characteristics	POD (*n* = 41)	Non‐POD (*n* = 147)	*p* Value
Age (year)	57 (55, 60)	58 (56, 60)	0.159
Female (*n*, %)	27 (66)	110 (75)	0.253
BMI (kg/m^2^)	27.3 (23.7, 28.9)	26.7 (24.2, 28.7)	0.710
OA duration (year)	3.0 (1.0, 7.5)	4.0 (2.0, 6.0)	0.322
MMSE before TKA	25 (24, 27)	25 (24, 27)	0.779
Education ≥ 9 years (*n*, %)	7 (17)	11 (7)	0.076
K‐L grade (*n*, %)			
I	1(2)	5(3)	0.973
II	3(7)	13(9)	
III	30(73)	106(72)	
IV	7(17)	23(16)	
Hypertension (*n*, %)			
Yes	11(27)	36(24)	0.760
Smoking (*n*, %)			
Yes	22(54)	47(32)	0.011*
SHS (*n*, %)			
Yes	15(37)	66(45)	0.342
Drinking (*n*, %)			
Yes	6(15)	22(15)	0.958
HAD score	7 (5, 9)	6 (5, 8)	0.375
PCS score	31 (18, 37)	25 (16, 34)	0.145
Midazolam (mg)	1.0 (0.9, 1.0)	1.0 (0.9, 1.0)	0.442
Propofol (mg)	449.0 (438.5, 464.0)	453.0 (434.0,473.0)	0.540
Remifentanil (μg)	769.0 (727.0, 831.5)	772.0 (724.0, 833.0)	0.846
Sufentanil (μg)	44.0 (42.5, 47.0)	44.0 (41.0, 47.0)	0.363
Amount of bleeding (mL)	50 (50, 50)	50 (50, 50)	0.679
Time to apply the tourniquet (min)	90.0 (67.5, 91.5)	81.0 (62.0, 90.0)	0.102
Duration of surgery (min)	90.0 (82.5, 100.0)	93.0 (80.0, 105.0)	0.587
Hospital stays (day)	3 (3, 5)	3 (3, 3)	<0.001*

*Note:* Data are shown as number (%) or median (inter‐quartile range). The Mann–Whitney *U*‐tests to identify difference between two groups. Categorical data are analyzed using the chi‐square or Fisher's exact test.

Abbreviation: BMI, body mass index; HAD, hospital anxiety and depression; K‐L, Kellgren‐Lawrence; MMSE, Mini‐Mental State Examination; OA, osteoarthritis; PCS, pain catastrophizing scale; POD, postoperative delirium; SHS: secondhand smoke; TKA, total knee arthroplasty.

* The difference was statistically significant (*p* < 0.05).

### 3.2 POD and smoking

**FIGURE 2:**
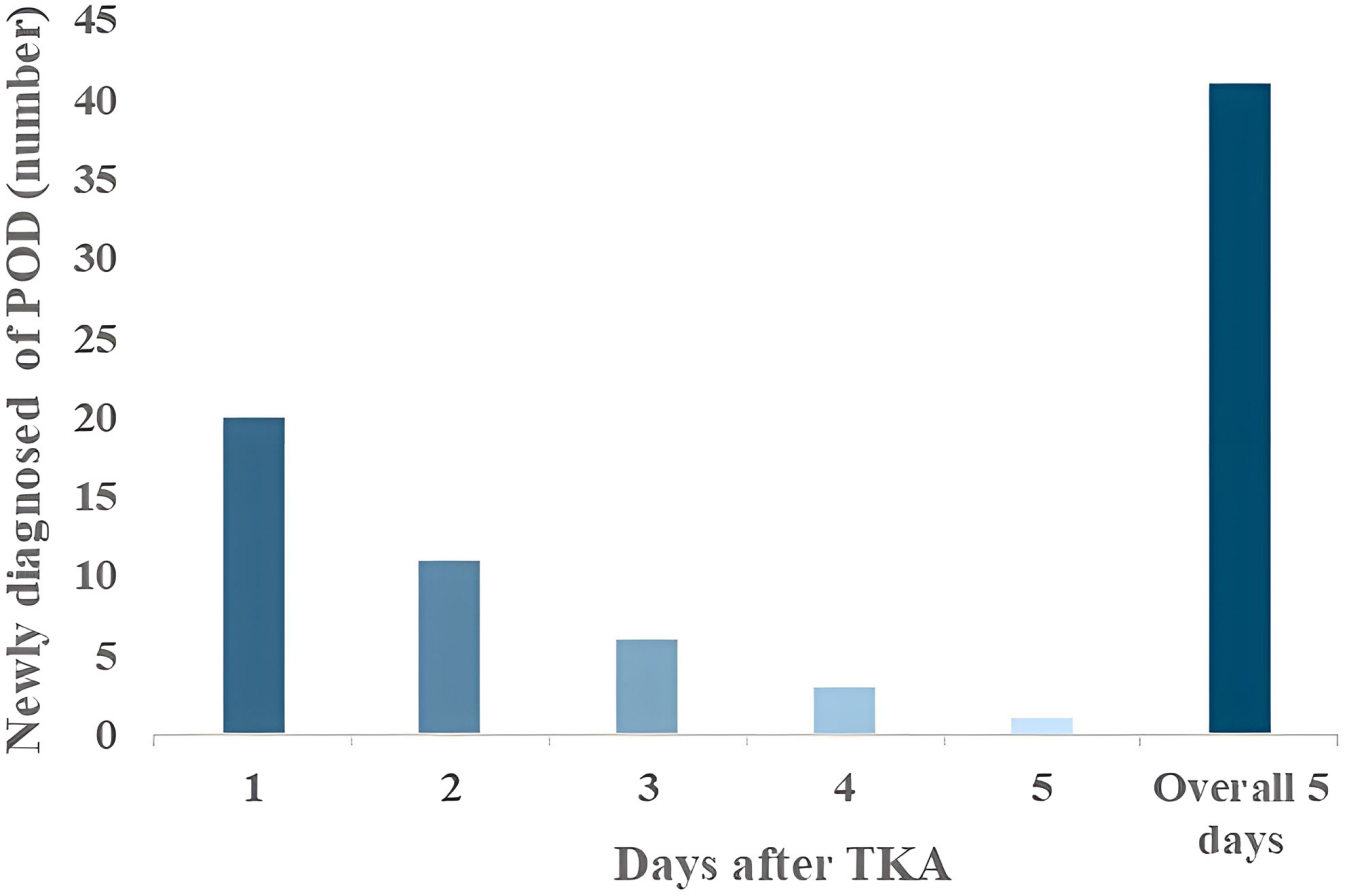
Newly diagnosed POD on postoperative days 1, 2, 3, 4 and 5 and overall. POD, postoperative delirium; TKA, total knee arthroplasty.

**FIGURE 3:**
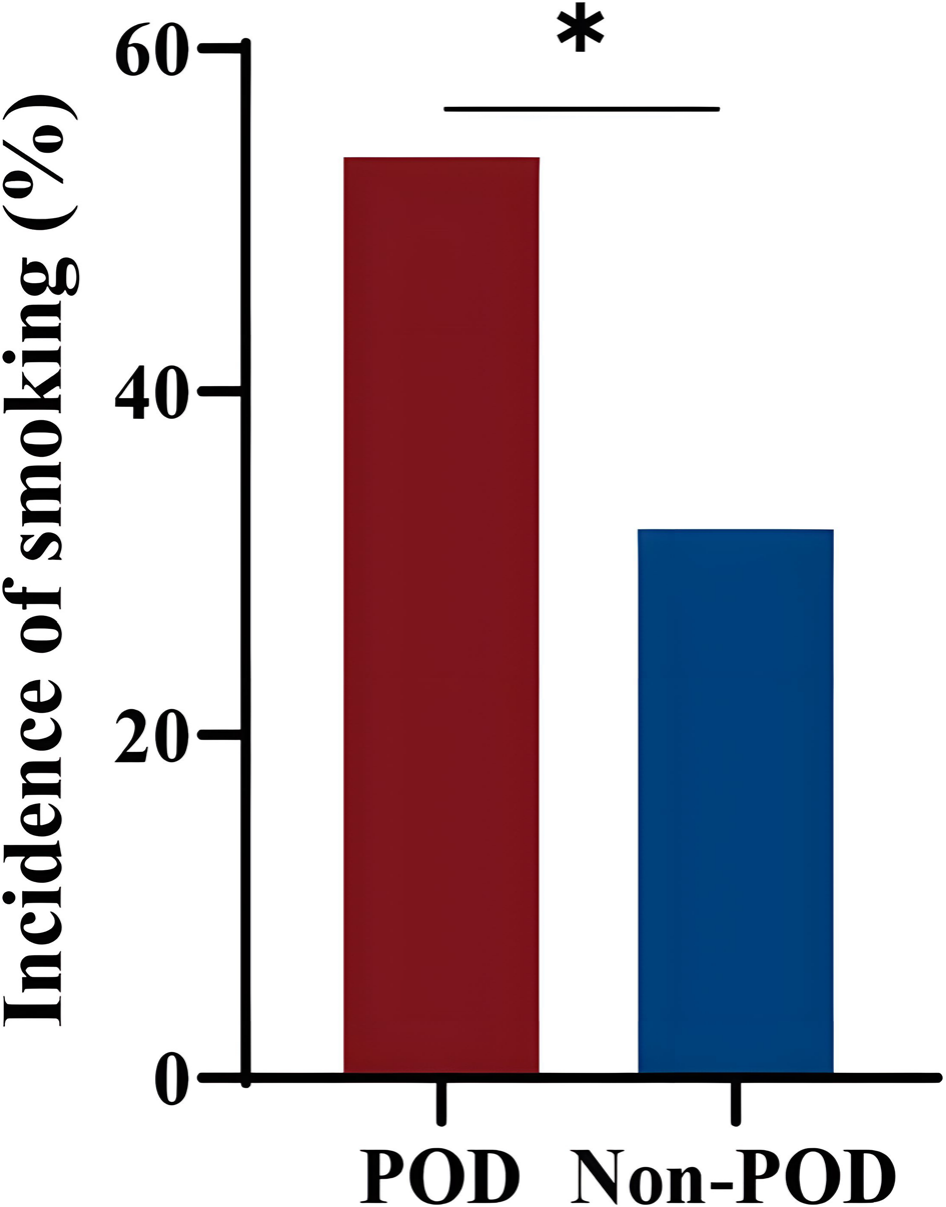
Comparison of smoking rates between the two groups. All data are presented as *n* (%), *p < 0.05 vs. Group Non‐POD. POD, postoperative delirium.

**FIGURE 4:**
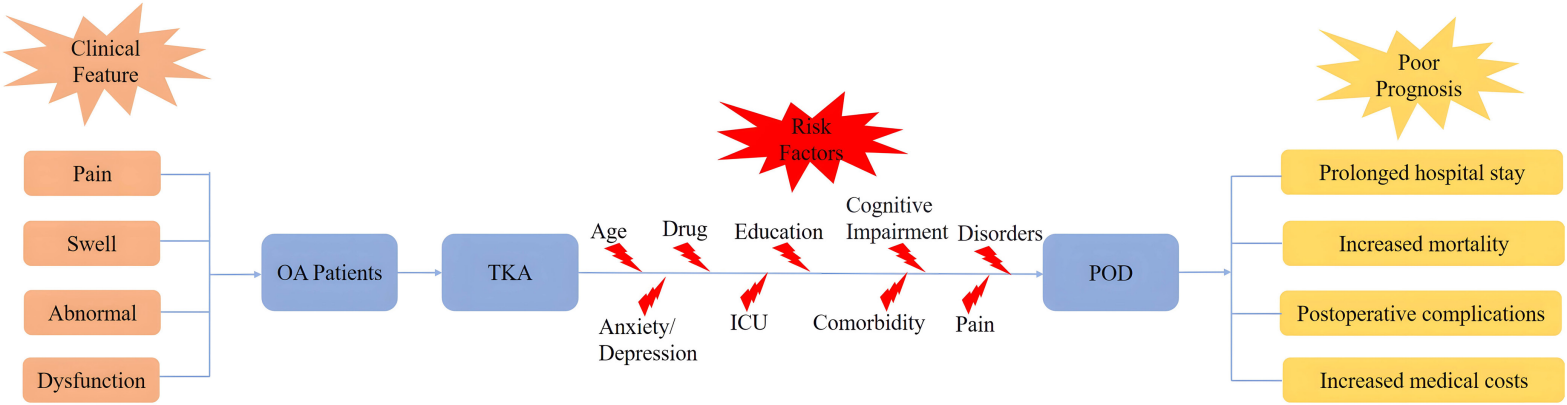
Clinical manifestations of OA, risk factors for developing POD and poor prognosis after undergoing TKA. OA, osteoarthritis; POD, postoperative delirium; TKA, total knee arthroplasty.

Forty‐one (21.8%) of 188 patients developed POD. POD was newly diagnosed 1 day (*n* = 20, 48.8%), 2 days (*n* = 11, 26.8%), 3 days (*n* = 6, 14.6%), 4 days (*n* = 3, 7.3%) and 5 days (*n* = 1, 2.4%) after surgery, with fewer patients developing POD as time progressed (Figure 2). In this study, former smokers accounted for only two of the participants, and they did not develop POD. Therefore, “current smokers” were judged as smokers, “former smokers” and “never smokers” were judged as non‐smokers. The incidence of smoking was significantly higher in Group POD than in Group Non‐POD (54 vs. 32, *p* < 0.05; Figure 3). In the univariate analysis, there were significant differences in smoking, hospital stays and VAS scores at rest on the second day after surgery between the two groups (*p* < 0.05). Based on previously published literature^26^ and clinical experience, some characteristics such as age, education, pain, anxiety, depression, comorbidities (hypertension, diabetes and stroke), drinking, drug use and ICU admission were considered as risk factors for the development of POD (Figure 4). Pain is one of the main clinical manifestations of OA patients and a primary reason for undergoing TKA. Smoking frequently occurs in chronic pain conditions, such as OA, and has been identified as a risk factor for pain.^14^ Preoperative pain is associated with a 1.5‐ to 3‐fold higher risk of POD.^27^, ^28^ Additionally, the postoperative pain score positively correlates with the risk of POD.^29^, ^30^ VAS scores at different time points in both groups are shown in Table 2. In this study, only two patients were admitted to the ICU after surgery and returned to the orthopedic ward 12 h later, so ICU admission was not included in the multivariate analysis. Finally, the risk factors associated with the occurrence of POD were included in the statistics, and binary logistic regression analysis was performed. The results in Table 3 show that patients who smoked preoperatively had an increased risk of developing POD (OR: 4.018, 95% CI: 1.158–13.947, *p* = 0.028). Patients in Group POD had a longer hospital stay than those in Group Non‐POD, which is consistent with other reports (OR: 3.469, 95% CI: 1.836–6.556, *p* < 0.001).

**TABLE 2:** Pain scores at different time points in both groups (*n* = 188).

VAS Score	POD (*n* = 41)	Non‐POD (*n* = 147)	*p* Value
At rest, cm			
Preoperative	2 (1, 2)	1 (1, 2)	0.446
Postoperative day1	1 (1, 2)	1 (1, 2)	0.152
Postoperative day2	2 (1, 2)	2 (1, 2)	0.033*
Postoperative day3	2 (2, 3)	2 (2, 3)	0.772
During movement, cm			
Preoperative	4 (3, 4)	4 (3, 4)	0.750
Postoperative day1	4 (2, 4)	3 (2, 4)	0.272
Postoperative day2	4 (3, 4)	3 (3, 4)	0.398
Postoperative day3	4 (3, 5)	4 (3, 5)	0.519

*Note:* Data are shown as median (inter‐quartile range). The Mann–Whitney *U*‐tests are used to identify difference between two groups.

Abbreviation: POD, postoperative delirium; VAS, visual analogue scale.

* The difference was statistically significant (*p* < 0.05).

**TABLE 3:** Logistic regression analysis to identify factors that predicted POD (*n* = 188).

Variables	*β*	OR	95%CI	*p* Value
Age	−0.063	0.939	0.841–1.048	0.259
Education	0.727	2.069	0.597–7.169	0.251
Preoperative VAS				
At rest	−0.089	0.915	0.466–1.795	0.796
During movement	0.050	1.052	0.590–1.876	0.865
Postoperative days 1 to 3				
Average VAS at rest	0.560	1.751	0.736–4.166	0.205
Average VAS during movement	0.202	1.224	0.680–2.205	0.500
HAD ≥ 8 scores	0.552	1.736	0.237–12.716	0.587
PCS ≥ 30 scores	0.150	1.162	0.491–2.753	0.733
Hypertension	0.342	1.408	0.525–3.788	0.496
Smoking	1.391	4.018	1.158–13.947	0.028*
SHS	0.572	1.772	0.493–6.372	0.381
Drinking	−0.140	0.869	0.276–2.739	0.811
Midazolam	−1.698	0.183	0.009–3.765	0.271
Remifentanil	−0.001	1.000	0.994–1.006	0.920
Sufentanil	0.024	1.024	0.908–1.155	0.700
Hospital stays	1.244	3.469	1.836–6.556	<0.001*

Abbreviation: HAD, hospital anxiety and depression; SHS: secondhand smoke; VAS, visual analogue scale.

* The difference was statistically significant (*p* < 0.05).

### 3.3 Other outcomes

Eighteen patients developed nausea or vomiting. Four patients required blood transfusions. Three patients developed postoperative hypothermia. Two patients were admitted to the ICU postoperatively due to difficulty in removing the tracheal tube. In Table 4, the results show no significant difference. None of the patients developed wound infections or died after surgery.

**TABLE 4:** Other outcomes of patients (*n* = 188).

	POD (*n* = 41)	Non‐POD (*n* = 147)	*p* Value
Rescue analgesia, *n* (%)	3 (7)	18 (12)	0.545
Nausea/vomiting, *n* (%)	3 (7)	15 (10)	0.798
Blood transfusion, *n* (%)	1 (2)	3 (2)	0.630
Hypothermia, *n* (%)	1 (2)	2 (1)	0.524
Admitted to ICU, *n* (%)	1 (2)	1 (0.6)	0.390
Incision infection, *n* (%)	0	0	–

*Note:* Data are presented as number (%). Categorical data are presented as frequencies and analyzed using the chi‐square or Fisher's exact test.

Abbreviation: ICU, intensive care unit; POD, postoperative delirium.

## 4 DISCUSSION

Despite the increasing success rate of TKA surgery, there are still patients who express dissatisfaction due to a series of postoperative complications, such as POD, which can delay rehabilitation and hospital discharge.^6^ Smoking may represent a potentially modifiable risk factor for developing POD in patients with TKA. Considering the high prevalence of smoking among patients with OA, it is of great significance for smoking cessation or nicotine replacement in perioperative management in the clinic. Decreased acetylcholine and increased dopamine levels are thought to be important factors in this process of delirium, although the exact pathophysiological process of POD is unclear.^31^ Similarly, in the pathophysiological process of nicotine withdrawal, the relative deficiency of acetylcholine also plays a central role. The sudden cessation of nicotine stimulation in smokers before surgery may be associated with POD.^32^ In fact, nicotine withdrawal and delirium share common characteristics, including depression, insomnia, irritability/frustration/anger, restlessness, loss of concentration, fidgeting, decreased heart rate, increased appetite or weight gain.^33^ Furthermore, nicotine withdrawal symptoms peak in the first week and can persist for up to 2–4 weeks after cessation, overlapping with the onset of POD,^34^ which is usually diagnosed upon admission to the ICU or several days postoperatively.^35^ Therefore, to reduce perioperative nicotine withdrawal symptoms, nicotine replacement therapy (NRT) has long been recognized and recommended for smokers.^36^

A recent study showed a relationship between increasing years of smoking and pain severity/frequency.^37^ This co‐occurrence of smoking behavior among patients suffering osteoarthritic pain is thought to be a result of reciprocal interactions in the manner of a positive feedback loop, leading to increased pain and cigarette smoking dependence.^38^ Smoking reduces pain for a short period of time, which in turn reinforces smoking behavior.^39^ On the other hand, anxiety/depression symptoms are emotional factors that regulate pain response and are also associated with cigarette smoking dependence.^40^ Increased catecholamine release due to anxiety may be the cause of peripheral sensitization, which leads to nociception.^41^ The positive correlation between pain‐related anxiety and cigarette smoking dependence may be one reason why smoking is more prevalent in patients with osteoarthritic pain and why smokers with chronic pain are more likely to be nicotine dependent. It seems to be a cycle where anxiety leads to smoking and then people become addicted to nicotine and experience acute withdrawal symptoms similar to anxiety. Smoking increases the risk of anxiety and exacerbates its severity.^33^

**FIGURE 5:**
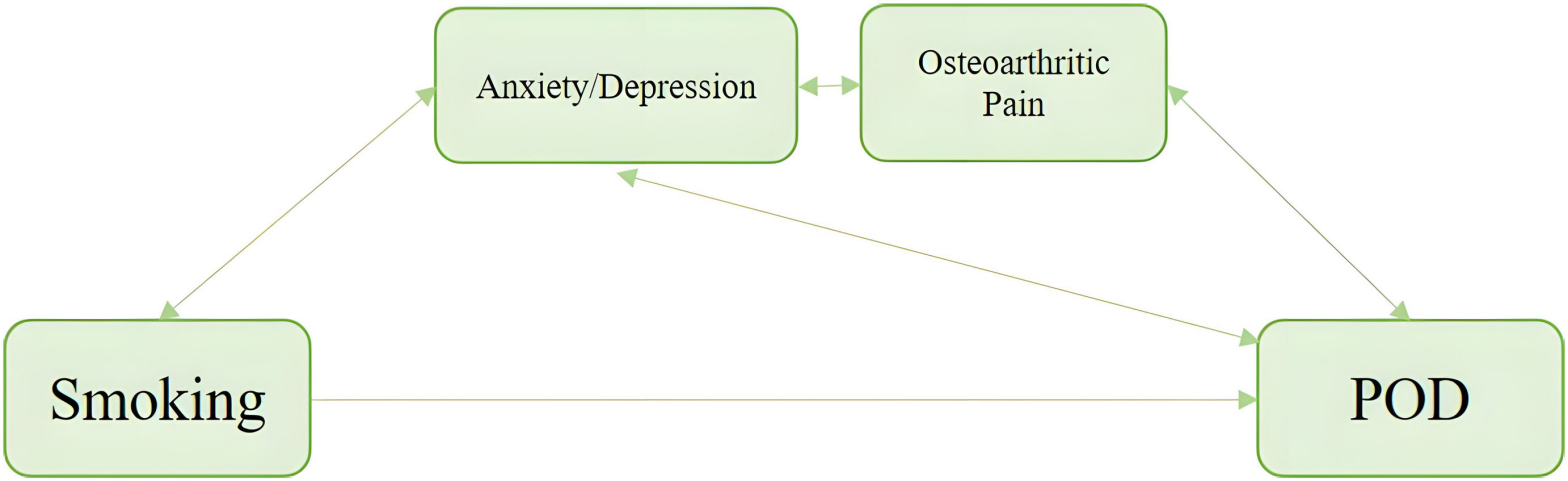
Integrative reciprocal model of smoking and POD in chronic pain, such as osteoarthritic pain. POD, postoperative delirium.

It has been previously shown that pain and anxiety/depression are risk factors for POD (Figure 5). Delirium is a debilitating neuropsychological disorder caused by a variety of stressors, including infections, drug toxicity and metabolic abnormalities, which can lead to an acute stress response in the brain. Pain has a similar effect, dramatically inducing catecholamine release and producing a pro‐inflammatory sympathetic response for a short period of time. Dysfunction of the cortisol system and chronic hyperactivity of inflammatory cytokines may be affected by chronic pain. Thus, acute or chronic pain may increase the chance of delirium.^42^ Preoperative depression is common in patients with delirium, and depression is a recognized sequel in patients with delirium. Depression and delirium share similar risk factors and pathophysiological mechanisms, including disturbed physiological responses and monoaminergic and melatonin function.^43^ However, we did not conclude that pain or anxiety/depression influenced the association between smoking and delirium in this study by mediating analysis by linear regression.

Some researchers have found that nicotine acts as a double‐edged sword in terms of cognitive function. Cognitive enhancement does not usually occur under smoking conditions but rather during exposure to nicotine. In animal studies, nicotine has been shown to improve motor and memory disorders in Parkinson's disease (PD) and Alzheimer's disease (AD).^44^, ^45^, ^46^ On the other hand, in human studies, cognitive function could be improved by nicotine supplementation and short‐term (<2 h) nicotine stimulation.^47^, ^48^ However, it has been suggested that the mechanism of impaired cognitive function may be related to nicotinic acetylcholine receptor desensitization.^49^

In this study, the POD incidence was 21.8%, similar to the results of previous studies.^10^ The prevalence of smoking among POD patients was 54%, more than half of the people who developed POD. We included the risk factors that have been previously shown to be associated with POD in the analysis. After excluding age, education, pain, anxiety, depression, hypertension, drinking, drug use and hospital stays, smoking was a risk factor for POD in patients with TKA. Smokers have a 4.018‐fold greater risk of developing POD than non‐smokers. Hospital stays were also significantly different. The length of hospital stay was correlated with the occurrence of POD. The occurrence of POD prolongs hospital stay. The mediating mechanism between smoking and POD needs to be studied. Does the incidence of POD after TKA decrease if a patient with knee OA has a history of smoking and quits for 2–4 weeks or longer before surgery? The duration of smoking cessation needs to be further determined.

However, some limitations should also be considered in this study. First, this study is a single‐center study. To reduce bias and eliminate relevant interfering factors as much as possible, our results need to be demonstrated by multi‐center research. Second, the amount and duration of cigarettes were not studied, so we did not conclude that the odds of POD were higher in patients who smoked for a long time/had a higher volume of smoking. Finally, this study was limited to patients with knee OA and cannot be extended to other knee conditions, such as rheumatoid arthritis. To reach a more accurate conclusion, the included diagnostic criteria should be expanded, and further studies should be conducted.

## 5 CONCLUSIONS

Collectively, OA patients who smoke have a higher incidence of POD after TKA, and the co‐occurrence of smoking behavior in OA patients has clinical relevance to the neuropsychological outcome after TKA.

## AUTHOR CONTRIBUTIONS

Conception and design of the study: DW and XQC. Literature search and collection and compilation of pictures and videos: JRC and JQC. Acquisition, analysis and interpretation of data: JCH, RSH, LS and HG. Drafting the article and revising it critically for important intellectual content: JRC, JQC, DW and XQC. Final approval of the article: all authors. JRC and JQC contributed equally to this work.

## FUNDING INFORMATION

There is no applicable funding for this study.

## CONFLICT OF INTEREST STATEMENT

The authors declare that there are no conflicts of interest.

## Data Availability

The data that support the findings of this study are available on request from the corresponding author. The data are not publicly available due to privacy or ethical restrictions.

## References

[1] Birch S , Stilling M , Mechlenburg I , Hansen TB . The association between pain catastrophizing, physical function and pain in a cohort of patients undergoing knee arthroplasty. BMC Musculoskel Dis. 2019;20:421. 10.1186/s12891-019-2787-6 PMC6739909 31511076

[2] Katz JN , Arant KR , Loeser RF . Diagnosis and treatment of hip and knee osteoarthritis: a review. J Am med Assoc. 2021;325:568‐578. 10.1001/jama.2020.22171 PMC8225295 33560326

[3] Dieppe PA , Lohmander LS . Pathogenesis and management of pain in osteoarthritis. Lancet. 2005;365:965‐973. 15766999 10.1016/S0140-6736(05)71086-2

[4] Lo C , Tsang W , Yan CH , Lord SR , Hill KD , Wong A . Risk factors for falls in patients with total hip arthroplasty and total knee arthroplasty: a systematic review and meta‐analysis. Osteoarthr Cartilage. 2019;27:979‐993. 10.1016/j.joca.2019.04.006 31028883

[5] Kurtz S , Ong K , Lau E , Mowat F , Halpern M . Projections of primary and revision hip and knee arthroplasty in the United States from 2005 to 2030. J Bone Joint Surg Am. 2007;89:780‐785. 17403800 10.2106/JBJS.F.00222

[6] Meyer M , Gotz J , Parik L , et al. Postoperative delirium is a risk factor for complications and poor outcome after total hip and knee arthroplasty. Acta Orthop. 2021;92:695‐700. 34607501 10.1080/17453674.2021.1980676 PMC8635535

[7] Shen L , Chen JQ , Yang XL , et al. Flurbiprofen used in one‐lung ventilation improves intraoperative regional cerebral oxygen saturation and reduces the incidence of postoperative delirium. Front Psychiatr. 2022;13:889637. 10.3389/fpsyt.2022.889637 PMC9470861 36117654

[8] Lee KH , Ha YC , Lee YK , Kang H , Koo KH . Frequency, risk factors, and prognosis of prolonged delirium in elderly patients after hip fracture surgery. Clin Orthop Relat R. 2011;469:2612‐2620. 10.1007/s11999-011-1806-1 PMC3148394 21327416

[9] Bin ARH , Yung WY . Postoperative delirium in patients undergoing Total joint arthroplasty: a systematic review. J Arthroplasty. 2015;30:1414‐1417. 25818653 10.1016/j.arth.2015.03.012

[10] Zhou Q , Zhou X , Zhang Y , et al. Predictors of postoperative delirium in elderly patients following total hip and knee arthroplasty: a systematic review and meta‐analysis. BMC Musculoskel Dis. 2021;22:945. 10.1186/s12891-021-04825-1 PMC8588632 34772392

[11] Ma J , Li C , Zhang W , et al. Preoperative anxiety predicted the incidence of postoperative delirium in patients undergoing total hip arthroplasty: a prospective cohort study. BMC Anesthesiol. 2021;21:48. 33579195 10.1186/s12871-021-01271-3 PMC7879687

[12] Ren A , Zhang N , Zhu H , Zhou K , Cao Y , Liu J . Effects of preoperative anxiety on postoperative delirium in elderly patients undergoing elective orthopedic surgery: a prospective observational cohort study. Clin Interv Aging. 2021;16:549‐557. 33814900 10.2147/CIA.S300639 PMC8009348

[13] Van Grootven B , Detroyer E , Devriendt E , et al. Is preoperative state anxiety a risk factor for postoperative delirium among elderly hip fracture patients? Geriatr Gerontol Int. 2016;16:948‐955. 26271367 10.1111/ggi.12581

[14] Felson DT , Zhang Y . Smoking and osteoarthritis: a review of the evidence and its implications. Osteoarthr Cartilage. 2015;23:331‐333. 10.1016/j.joca.2014.11.022 PMC5473429 25454371

[15] Kong L , Wang L , Meng F , Cao J , Shen Y . Association between smoking and risk of knee osteoarthritis: a systematic review and meta‐analysis. Osteoarthr Cartilage. 2017;25:809‐816. 10.1016/j.joca.2016.12.020 28011100

[16] Johnsen MB , Pihl K , Nissen N , et al. The association between smoking and knee osteoarthritis in a cohort of Danish patients undergoing knee arthroscopy. BMC Musculoskel dis. 2019;20:141. 10.1186/s12891-019-2518-z PMC6444863 30935365

[17] Leventhal AM , Zvolensky MJ . Anxiety, depression, and cigarette smoking: a transdiagnostic vulnerability framework to understanding emotion‐smoking comorbidity. Psychol Bull. 2015;141:176‐212. 25365764 10.1037/bul0000003 PMC4293352

[18] Moylan S , Jacka FN , Pasco JA , Berk M . How cigarette smoking may increase the risk of anxiety symptoms and anxiety disorders: a critical review of biological pathways. Brain Behav. 2013;3:302‐326. 23785661 10.1002/brb3.137 PMC3683289

[19] He Y , Lam TH , Jiang B , et al. Passive smoking and risk of peripheral arterial disease and ischemic stroke in Chinese women who never smoked. Circulation. 2008;118:1535‐1540. 18809795 10.1161/CIRCULATIONAHA.108.784801

[20] Wu SY , Xing F , Sharma S , et al. Nicotine promotes brain metastasis by polarizing microglia and suppressing innate immune function. J Exp med. 2020;217:e20191131. 32496556 10.1084/jem.20191131 PMC7398164

[21] Wei LA , Fearing MA , Sternberg EJ , Inouye SK . The confusion assessment method: a systematic review of current usage. J Am Geriatr Soc. 2008;56:823‐830. 18384586 10.1111/j.1532-5415.2008.01674.x PMC2585541

[22] Inouye SK , van Dyck CH , Alessi CA , Balkin S , Siegal AP , Horwitz RI . Clarifying confusion: the confusion assessment method: A new method for detection of delirium. Ann Intern med. 1990;113:941‐948. 2240918 10.7326/0003-4819-113-12-941

[23] Wang J , Mao G , Malackany N , et al. Association between perioperative hypotension and postoperative delirium and atrial fibrillation after cardiac surgery: a post‐hoc analysis of the decade trial. J Clin Anesth. 2022;76:110584. 34784557 10.1016/j.jclinane.2021.110584

[24] Cui F , Zhao W , Mu DL , et al. Association between cerebral desaturation and postoperative delirium in thoracotomy with one‐lung ventilation: a prospective cohort study. Anesth Analg. 2021;133:176‐186. 33721874 10.1213/ANE.0000000000005489

[25] Snaith RP , Zigmond AS . The hospital anxiety and depression scale. Br med J. 1986;292:344. 10.1136/bmj.292.6516.344 PMC1339318 3080166

[26] Oh ST , Park JY . Postoperative delirium. Korean J Anesthesiol. 2019;72:4‐12. 30139213 10.4097/kja.d.18.00073.1 PMC6369344

[27] Kosar CM , Tabloski PA , Travison TG , et al. Effect of preoperative pain and depressive symptoms on the development of postoperative delirium. Lancet Psychiat. 2014;1:431‐436. 10.1016/S2215-0366(14)00006-6 PMC4307613 25642413

[28] Tan MC , Felde A , Kuskowski M , et al. Incidence and predictors of post‐cardiotomy delirium. Am J Geriat Psychiat. 2008;16:575‐583. 10.1097/JGP.0b013e318172b418 18591577

[29] Jin Z , Hu J , Ma D . Postoperative delirium: perioperative assessment, risk reduction, and management. Brit J Anaesth. 2020;125:492‐504. 32798069 10.1016/j.bja.2020.06.063

[30] Xue P , Wu Z , Wang K , Tu C , Wang X . Incidence and risk factors of postoperative delirium in elderly patients undergoing transurethral resection of prostate: a prospective cohort study. Neuropsych Dis Treat. 2016;12:137‐142. 10.2147/NDT.S97249 PMC4716723 26834475

[31] Maldonado JR . Neuropathogenesis of delirium: review of current etiologic theories and common pathways. Am J Geriat Psychiat. 2013;21:1190‐1222. 10.1016/j.jagp.2013.09.005 24206937

[32] Skrobik Y . Broadening our perspectives on ICU delirium risk factors. Crit Care. 2009;13:160. 19591660 10.1186/cc7917 PMC2750136

[33] Parkerson HA , Zvolensky MJ , Asmundson GJ . Understanding the relationship between smoking and pain. Expert Rev Neurother. 2013;13:1407‐1414. 24236905 10.1586/14737175.2013.859524

[34] Hughes JR . Tobacco withdrawal in self‐quitters. J Consult Clin Psych. 1992;60:689‐697. 10.1037//0022-006x.60.5.689 1401384

[35] Hsieh SJ , Shum M , Lee AN , Hasselmark F , Gong MN . Cigarette smoking as a risk factor for delirium in hospitalized and intensive care unit patients. A systematic review. Ann Am Thorac Soc. 2013;10:496‐503. 24161052 10.1513/AnnalsATS.201301-001OC PMC3960910

[36] Stead LF , Perera R , Bullen C , et al. Nicotine replacement therapy for smoking cessation. Cochrane Database Syst Rev. 2012;11:D146. 10.1002/14651858.CD000146.pub4 23152200

[37] Weingarten TN , Iverson BC , Shi Y , Schroeder DR , Warner DO , Reid KI . Impact of tobacco use on the symptoms of painful temporomandibular joint disorders. Pain. 2009;147:67‐71. 19793624 10.1016/j.pain.2009.08.021

[38] Ditre JW , Brandon TH , Zale EL , Meagher MM . Pain, nicotine, and smoking: research findings and mechanistic considerations. Psychol Bull. 2011;137:1065‐1093. 21967450 10.1037/a0025544 PMC3202023

[39] Hahn EJ , Rayens MK , Kirsh KL , Passik SD . Brief report: pain and readiness to quit smoking cigarettes. Nicotine Tob Res. 2006;8:473‐480. 16801305 10.1080/14622200600670355

[40] Ditre JW , Zale EL , Kosiba JD , Zvolensky MJ . A pilot study of pain‐related anxiety and smoking‐dependence motives among persons with chronic pain. Exp Clin Psychopharm. 2013;21:443‐449. 10.1037/a0034174 PMC3935323 24080021

[41] Smit T , Peraza N , Garey L , et al. Pain‐related anxiety and smoking processes: the explanatory role of dysphoria. Addict Behav. 2019;88:15‐22. 30103097 10.1016/j.addbeh.2018.08.008 PMC10062193

[42] Hannibal KE , Bishop MD . Chronic stress, cortisol dysfunction, and pain: a psychoneuroendocrine rationale for stress management in pain rehabilitation. Phys Therapy. 2014;94:1816‐1825. 10.2522/ptj.20130597 PMC4263906 25035267

[43] Pakrad F , Pakrad E , Darvishi N , Poorolajal J . Preoperative anxiety and depression increases the incidence of delirium after coronary artery bypass graft surgery. J Perianesth Nurs. 2020;35:496‐501. 32499109 10.1016/j.jopan.2020.01.017

[44] Alhowail A . Molecular insights into the benefits of nicotine on memory and cognition (review). Mol med Rep. 2021;23:398. 33786606 10.3892/mmr.2021.12037 PMC8025477

[45] Nicholatos JW , Francisco AB , Bender CA , et al. Nicotine promotes neuron survival and partially protects from Parkinson's disease by suppressing SIRT6. Acta Neuropathol Com. 2018;6:120. 10.1186/s40478-018-0625-y PMC6223043 30409187

[46] Giese KP , Mizuno K . The roles of protein kinases in learning and memory. Learn Mem. 2013;20:540‐552. 24042850 10.1101/lm.028449.112

[47] Valentine G , Sofuoglu M . Cognitive effects of nicotine: recent progress. Curr Neuropharmacol. 2018;16:403‐414. 29110618 10.2174/1570159X15666171103152136 PMC6018192

[48] Heishman SJ , Kleykamp BA , Singleton EG . Meta‐analysis of the acute effects of nicotine and smoking on human performance. Psychopharmacology (Berl). 2010;210:453‐469. 20414766 10.1007/s00213-010-1848-1 PMC3151730

[49] Levin ED . Complex relationships of nicotinic receptor actions and cognitive functions. Biochem Pharmacol. 2013;86:1145‐1152. 23928190 10.1016/j.bcp.2013.07.021 PMC3797209

